# Prevalence of Diabetes Type 2 in Hepatitis C Infected Patients in Kpk, Pakistan

**DOI:** 10.1155/2017/2416281

**Published:** 2017-04-04

**Authors:** Ghani Ur Rehman, Mohammad Ali, Farooq Shah, Amjad Iqbal, Ayaz Ahmad, Zafar Hayat, Badshah Islam, Farman Ali, Ikramullah, Yousaf Jamal, Sartaj Alam, Muhammad Sajjad, Muhammad Zeeshan Bhatti

**Affiliations:** ^1^Department of Biotechnology, Bacha Khan University, Charsadda, Pakistan; ^2^Department of Agriculture, Abdul Wali Khan University Mardan, Mardan, Pakistan; ^3^Coconut Research Institute, Chinese Academy of Tropical Agricultural Sciences, Wenchang City, Hainan 571339, China; ^4^Department of Biotechnology, Abdul Wali Khan University Mardan, Mardan, Pakistan; ^5^Institute of Genetics and Developmental Biology, Chinese Academy of Sciences, Beijing, China; ^6^Department of Plant Breeding and Genetics, Bacha Khan University, Charsadda, Pakistan; ^7^Department of Agriculture, University of Swabi, Ambar, Pakistan; ^8^Department of Pathology, The University of Agriculture, Peshawar, Pakistan; ^9^Institute of Biomedical Sciences, East China Normal University, Shanghai, China

## Abstract

Hepatitis C (HCV) and diabetes mellitus are the two main health concerns that cause devastating health and financial worries worldwide. It has been observed in the past that both diseases have a high correlation that might be due to the abnormal conditions of the liver. But the mechanism of the prevalence of diabetes in patients with chronic HCV infection still remains unclear. In our study, we have investigated T2DM in the male and female patients at Lady Reading Hospital (LRH), Peshawar. The blood samples of both in- and outpatients were analysed in the PCR laboratories of LRH from December 2014 to April 2015. Great prevalence of diabetes in hepatitis C infected male and female patients was observed during this study. The data were collected from the patients through a preplanned questionnaire that included name of the patient, HCV, being diabetic, age, gender, location, educational background, family history of the disease, other diseases, and any treatments if taken. The results of our study have found 26.42% prevalence of T2DM in HCV infected patients. So we conclude that HCV infection may be one of the reasons that could lead to T2DM.

## 1. Introduction

Hepatitis C virus (HCV) infection is a life threatening serious infection and one of the major causes of chronic liver disease [1]. In 1975 the HCV infection was recognized as a separate disease for the first time and was classified as non-A and non-B hepatitis. Subsequently, in 1989 after the advancement in molecular biology, the virus was verified as life threatening. Hepatitis C virus actually belongs to the* Hepacivirus* genus (in Greek, hepatos means liver) and is further divided into seven major genotypes (i.e., 1, 2, 3, 4, 5, 6, and 7). The highest prevalence of the Hepatitis C among the general population was recorded in Egypt, which was about 12% of the total population [2]. Also, the prevalence of the disease was found high in patients with 40 years of age or above [3]. It is estimated that about 2.2–3% of the total population of the globe are infected with HCV [4, 5].

Diabetes mellitus (DM) is another main health related issue among the developed and nondeveloped nations of the world. DM is actually a metabolic disorder that can lead to life threatening retinopathy, nephropathy, neuropathy, and heart diseases, respectively [6, 7]. Regarding the increasing trends of DM, 70% increase has been reported in developing nations compared to 47% in the developed nations of the world [8]. Previously, DM was related to the older age, but recently it has been observed high among youth as well [9]. Thus, the main reasons behind this might be the livelihood of the people in the particular area.

Currently, there is no well-organized system to monitor the general trend of HCV and diabetes in Pakistan and their coexistence. Health regulatory authorities are only organizing educational campaigns to develop awareness among the public from last few years [10], which is not enough. A study was, therefore, designed with the aim to know the possible links between DM and HCV to know whether HCV can make a person vulnerable to DM or vice versa. In our findings, we observed a prevalence of the DM in the HCV infected patients, which might reflect on their close association with each other in the developing nations. The exact mechanism of this relationship is still not very clear which needs more research to discover the fact behind this relationship.

## 2. Materials and Methods

This study was conducted in the PCR laboratory of the LRH, Peshawar. A total of 1295 patients have participated in the study with 212 patients being found positive. Both inpatients and outpatients were monitored for DM and HCV during the study. The demographic data were collected in the form of information on a preplanned questioner consisting of name, age, gender, location, educational background, family history of the disease and other related diseases, and previous treatments taken.

In our study, we did not include pregnant women, patients with a history or evidence of pancreatitis, pancreatic tumor, hepatic tumor cirrhosis, or other coexisting viral infections like hepatitis B. All the patients were tested for random blood sugar according to the new diagnostic criteria designed by ADA and WHO (as hypoglycemic with less than 60 mg/dL, normoglycemic with 126 mg/dL, and hyperglycemic with 200 mg/dL of blood glucose, resp.) [11]. The DM was checked by Strip method, where the HCV test was done by real time qPCR.

### 2.1. Sample Collection and Processing

The blood samples from patients were collected in the RNAase free gel tubes already numbered and scanned. The samples were then centrifuged at 6000*g* for 5 minutes.

### 2.2. Material Required

Materials required were the following: buffer RAV1 2x 35 mL, buffer RAW 2x 30 mL, buffer RAV3 (concentrated) 2x 125 mL, buffer RE 2x 5 mL, carrier DNA lyophilized 2x 1 mg, Ribo Virus columns 100, collection tubes (2) 300, ethanol 96–100%, biological cabinet, microcentrifuge tubes, sterile RNAse-free pipette tips with aerosol barrier, disposable gloves (powder-less), microcentrifuge (with rotor for 2 mL tubes), and real time qPCR machine.

### 2.3. Extraction of DNA

1. 600 *μ*L of lysis buffer (RAVI) was taken in a tube and 150 *μ*L of blood serum was added to it. The contents were vortexed for 15 seconds and incubated for 5 minutes at 70°C.
2. After incubation, an equal volume of ethanol (96–100%) was added to the sample and the contents were mixed by using vortex for 15 seconds.
3. The samples were then transferred to new 2 mL centrifuge tubes and were centrifuged for a minute at 8000*g*. The sample (700 *μ*L) was then loaded onto a column.
4. The samples adsorbed onto the column were first washed thrice with 2 mL of DEPC water. RAW buffer (500 *μ*L) was then used to wash the nucleic acids adsorbed on the column. The tube was centrifuged at 8000*g* for a minute.
5. Second washing buffer, RAV3 (600 *μ*L), was added and centrifuged at 8000*g* for a minute. A second round of washing was done with RAV3 (200 *μ*L). For the addition of the buffer, the tubes were centrifuged at high speed of 11000*g* for 5 minutes to make sure that all the impurities and ethanol have been removed.
6. In the final step the column was first incubated at 70°C for a minute with an open cap to remove any traces of ethanol. After incubation the tube containing column was placed in a new RNAase free tube and a 50 *μ*L preheated (70°C) RE buffer was loaded at the top of the column. The column was incubated for 2 minutes and centrifuged at 11000*g* for a minute. Pure viral nucleic acid was collected in the new RNAse-free tube that was stored at −20°C before further processing. On the next day the samples were taken for PCR analysis as described by Sarrazin et al. [12].

## 3. Results 

In this study, a total of 1311 cases were studied for HCV by real time qPCR, out of which 212 cases were found positive with 16.18% of the total population tested. The number of male population (57.75%) with HCV was higher than the female (42.93%). HCV infection among the various age groups was highly prevalent in the subjects with 30–40 years of age, whereas only 32 cases were recorded positive in the age group of 40–50 years. In the younger population of 20–30 years of age, 52 cases were recorded, but in all age groups the occurrence of HCV was higher in male than in female. Data regarding locality reflects on the high number of positive cases in rural areas as compared to the urban areas. Out of the total 60 cases from the cities, the number of HCV positive male subjects was 35 (58.34%) and female subjects was 25 (41.66%). On the other hand, 152 (71.70%) patients were found positive from the villages (56.58% males and 43.42% females). In the present study, educational background was also taken into consideration which showed a low number of cases in the educated society (43.40% cases). Again, the HCV cases were found high in males (75%) in relation to the females (25%). In contrast, the number of uneducated patients with HCV was 120 with 44.17% of recorded males and 55.83% of females (Table 1).

**Figure 1:**
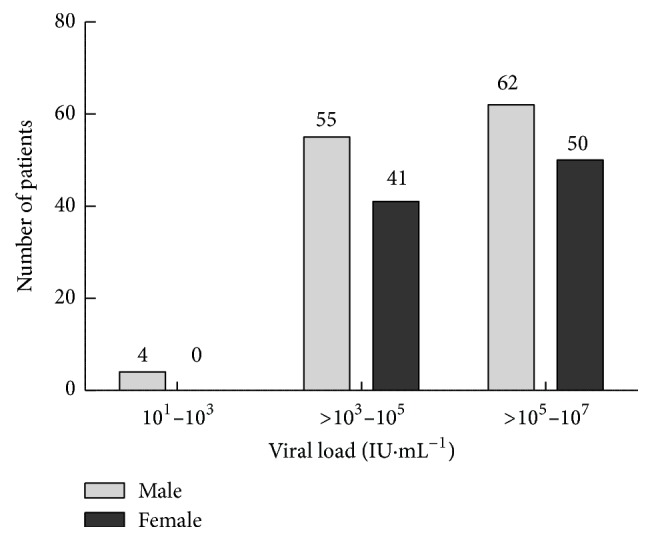
Incidence of HCV infection in male and female patients at LRH, Peshawar.

**Figure 2:**
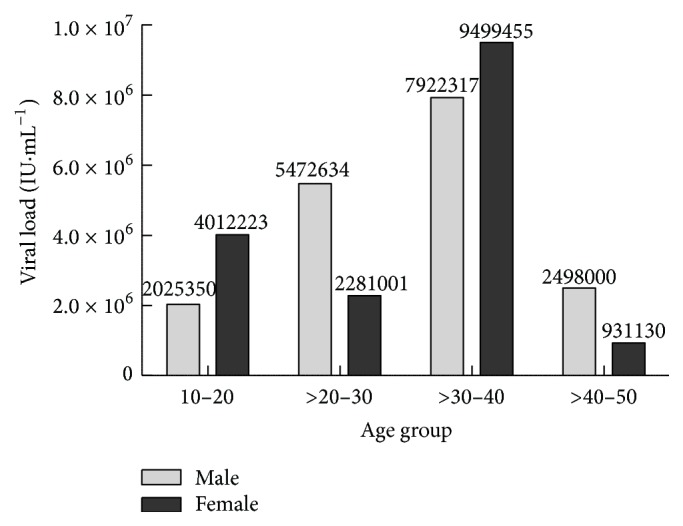
Magnitude of HCV infection in patients from various age groups.

**Figure 3:**
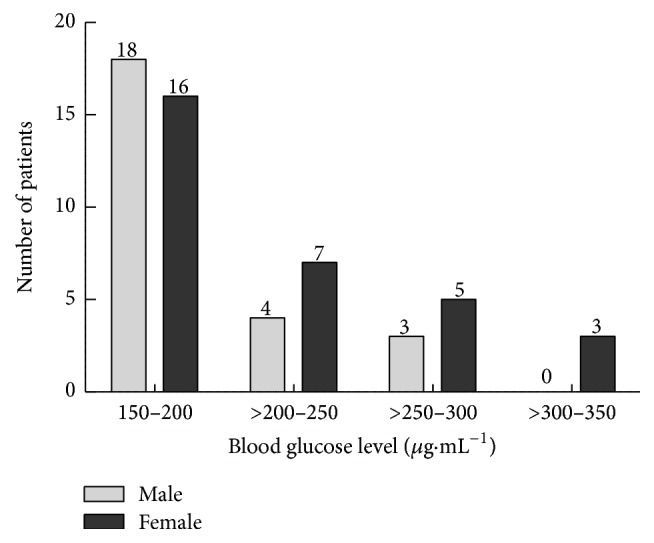
Prevalence of diabetes in male and female patients at LRH, Peshawar.

The overall viral load among the infected individuals was in the range of 1295 to 9500750 RNA IU mL^−1^; a lower ratio of viral load in male compared to the female was observed. The viral load among the male patients was in the range from 1333 to 7923983 RNA IU mL^−1^, whereas in female patients it was from 1295 to 9500750 RNA IU mL^−1^ (Figure 1). It was found interesting that both lower limit and upper limit of viral load were detected in female patients. A clear difference in the viral load was also witnessed amid the various age groups. The higher viral load was recorded in both male (7922371 RNA IU mL^−1^) and female (9499455 RNA IU mL^−1^) patients from 30–40 years age group. However, a low viral load was recorded in both genders from the 40–50 years of age group (Figure 2). The results in Figure 3 also revealed that almost one-third of the HCV patients were diabetic. Our data records showed that 26.42% of the HCV infected patients were found to have T2DM. The association of chronic HCV with liver cirrhosis was well established few years back, but with diabetes mellitus, it is quite recent.

## 4. Discussion

Several of the evidences from many studies have suggested a link between HCV infection and T2DM [13, 14]. On the basis of previous case controlled studies, the chances of prevalence of diabetes mellitus in HCV infected subjects are 21% to 50%. Various scientists had found a strong relationship between diabetes and HCV in HCV infected individuals. In one of the reports the prevalence of diabetes in HCV infection was 4.39 times higher than that in control group [15]. Similarly, in Sir Ganga Ram Hospital, Karachi, it was documented that diabetic patients, especially with T2DM, are at high risk of catching HCV infection [16]. Furthermore, it was estimated that about 42.3% patients with impaired glucose tolerance had HCV infection [17]. Thus, these studies made it a bit clear that the HCV patients in a community have to carry out regular tests of their blood glucose.

Interestingly, all the seropositive cases we have studied during our study were type II diabetics. Existing information about this association is very limited, which does not provide a solid proof for the mechanism of this relationship. From the history of patients, it was also noted that most of the patients have developed T2DM in less than five years of HCV infection which illustrates the role of HCV infection in the development of T2DM. It might be possible that HCV infection can affect the liver and pancreas of the affected individual, thus causing defects in the production of *β* cells resulting in high blood glucose levels. Also, the majority of the hepatitis C infected patients, who lately develop diabetes, was in the range of 30–40 years. Yet, in the United States, the coexistence of HCV infection and diabetes is common in aged people. It is quite logical because the older patients are the most vulnerable group for the extrahepatic manifestation of HCV infection.

## Figures and Tables

**Table 1:** Sociodemographic characteristics of HCV and diabetes.

Patients	Total	Male	Female
HCV positive	212	121	91
Age group: 20–30	52	30	22
Age group: 30–40	132	72	60
Age group: 40–50	28	19	9
Diabetic	56	25	31
Urban	60	35	25
Rural	152	86	66
Educated	92	69	23
Noneducated	120	53	67
